# Persistent Extended-Spectrum β-Lactamase Urinary Tract Infection

**DOI:** 10.3201/eid1511.081501

**Published:** 2009-11

**Authors:** Joan DeBusscher, Lixin Zhang, Miatta Buxton, Betsy Foxman, Cibele Barbosa-Cesnik

**Affiliations:** University of Michigan, Ann Arbor, Michigan, USA

**Keywords:** ESBL, Escherichia coli, community acquired, persistence, urinary tract infection, bacteria, letter

**To the Editor:** Uncomplicated urinary tract infections (UTIs) in otherwise healthy adults are usually treated empirically because the causative microbe is highly predictable: 80%–90% are caused by *Escherichia coli*. In addition, short courses of therapy (1 day or 3 days) are usually completed before laboratory results become available. In the past decade, reports of community-acquired, extended-spectrum β-lactamase (ESBL)–producing *E. coli* isolates have increased worldwide, but they are still uncommon in the United States (*1*), where reported cases are generally associated with hospitals. An early report of true community-acquired ESBL-producing *E. coli* infections in the United States was published in 2007 (*2*). We report a case of community-acquired lower UTI caused by ESBL-producing and multidrug resistant *E. coli* in an otherwise healthy college-aged woman who had no hospital exposure. Despite proper treatment, her infection persisted subclinically and symptoms recurred 2 months later.

The patient was an afebrile 24-year-old female college student who had visited her university health service, where she was recruited into a clinical trial investigating the effects of cranberry juice on UTIs. Inclusion in the study required that participants have UTI signs and symptoms, positive urine culture, and physician diagnosis. Participants provided self-collected vaginal, rectal, and midstream urine specimens at the time of enrollment and at 3- and 6-month follow-up or UTI recurrence. Study protocol was approved by the University of Michigan Institutional Review Board.

*E. coli* was isolated from all specimens collected from the patient at the time of enrollment; urinalysis confirmed pyuria (>100 leukocytes/high power field). Also at the time of enrollment, the patient reported no antimicrobial drug treatment during the previous 4 weeks, no history of hospitalization, no urethral catheterization, and no sexually transmitted infection (confirmed by medical record review). A 7-day regimen of nitrofurantoin was prescribed.

After 53 days, the patient returned to the health service with recurring UTI symptoms and was treated with a 3-day regimen of trimethoprim–sulfamethoxazole; no urine specimen was submitted at that time. However, *E. coli* isolates were recovered from recurrence urine and rectal specimens collected within 48 hours according to the clinical trial protocol. All *E. coli* isolates collected at the time of enrollment (n = 3) and recurrence (n = 2) appeared morphologically and phenotypically identical (API Rapid 20E; bioMérieux, Durham, NC, USA). Genotyping using enterobacterial repetitive intergenic consensus (ERIC) PCR with an ERIC-2 primer showed a shared ERIC type, indicating identity (Figure). When tested for antimicrobial drug susceptibility (Vitek 2; bioMérieux), all 5 isolates were identified as ESBL-producers and were resistant to β-lactams: ampicillin, cefazolin, ceftriaxone (MIC >64 µg/mL), aztreonam, and piperacillin. After an ESBL confirmatory test, recommended by the Clinical and Laboratory Standards Institute (*3*), showed positive results, the isolates were also considered resistant to ceftazidime (MIC 1–4 µg/mL) and cefepime. Disk diffusion indicated susceptibility to cefoxitin. The isolates were also resistant to fluoroquinolones, tetracycline, and trimethoprim–sulfamethoxazole but susceptible to aminoglycosides, carbapenems, and nitrofurantoin. Isolates from the time of enrollment had intermediate susceptibility to amoxicillin–clavulanate (MIC 16 µg/mL), but isolates from the recurrence episode were resistant (MIC 32 µg/mL).

**Figure:**
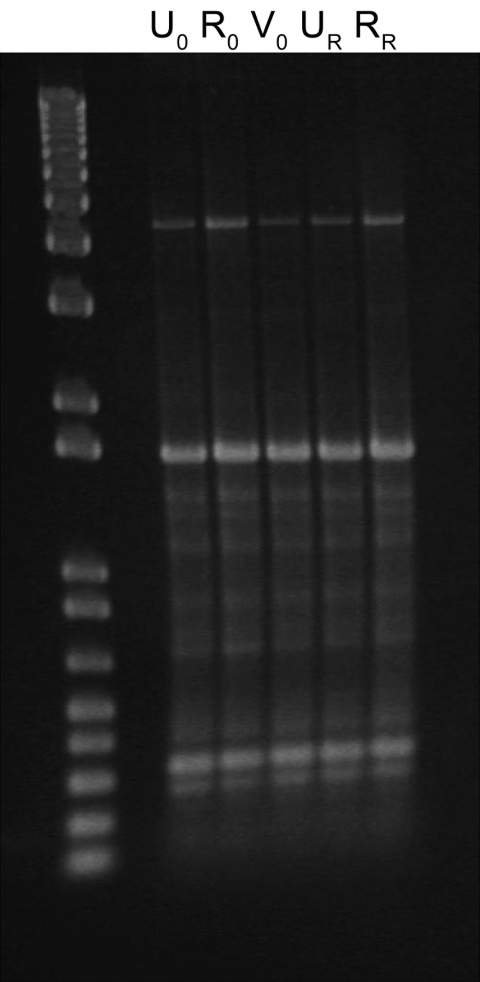
Enterobacterial repetitive intergenic consensus typing of extended-spectrum β-lactamase–producing *Escherichia coli* isolated from index (_0_) and recurring (_R_) urine (U), rectal (R), and vaginal (V) samples from a nonpregnant young woman.

Although the patient’s initial UTI was treated adequately with nitrofurantoin, the infection recurred, implying that it remained in a reservoir, not uncommon for uncomplicated UTIs (*4*,*5*). Alternative antimicrobial drug treatment for outpatients with ESBL-producing *Enterobacteriaceae* is limited. Carbapenems remain the most effective drugs (*6*) but must be administered intravenously or intramuscularly (*3*). The reported efficacy of fosfomycin (*7*) suggests an option, but because agar dilution is the only recommended testing method, use of this drug in the United States is hindered. Use of antimicrobial drugs that concentrate in urine remains controversial as long as resistance is interpreted by MIC (blood-level resistance).

PCR detected β-lactamase resistance genes in all isolates, identifying them as ESBL positive when CTX-M consensus primer PCR was used but negative with TEM and SHV. Sequence analysis of the amplified gene showed that it encoded a CTX-M-15–like ESBL.

This isolate’s increasing resistance to a β-lactamase–inhibitor combination, amoxicillin–clavulanate, suggested the possibility of inducible AmpC β-lactamase production. A negative AmpC disk test (with Tris/EDTA, cefoxitin, and *E. coli* ATCC 25922) refuted a plasmid-mediated AmpC β-lactamase (*6*); the remaining possible resistance mechanisms were hyperproduction of β-lactamase or an inhibitor-resistant penicillinase.

For the patient reported here, the multiple drug–resistant strain persisted for at least 53 days despite appropriate treatment with antimicrobial drugs. Furthermore, medical record review found an additional UTI caused by *E. coli* 12 weeks later. Thus, because of the long duration of carriage of this highly resistant strain, potential for transmission to others is high.

The low number of previous reports of community-acquired ESBL in the United States does not necessarily suggest low community prevalence. Reports of ESBL-producer bacteremia in patients visiting emergency rooms suggests earlier and wider incidence (*8*). Returning to the practice of regularly culturing urine samples is difficult to justify; however, without ongoing surveillance to detect and control ESBL resistance, prevalence can only be expected to rise.
